# Maternal blood cadmium, lead and arsenic levels, nutrient combinations, and offspring birthweight

**DOI:** 10.1186/s12889-017-4225-8

**Published:** 2017-04-24

**Authors:** Yiwen Luo, Lauren E. McCullough, Jung-Ying Tzeng, Thomas Darrah, Avner Vengosh, Rachel L. Maguire, Arnab Maity, Carmen Samuel-Hodge, Susan K. Murphy, Michelle A. Mendez, Cathrine Hoyo

**Affiliations:** 1 Bioinformatics Research Center, North Carolina State University, Raleigh, NC USA; 2 Department of Epidemiology, Emory University, Atlanta, GA USA; 3 Department of Statistics, North Carolina State University, Raleigh, NC USA; 4 Department of Statistics, National Cheng-Kung University, Tainan, Taiwan; 5 School of Earth Sciences, The Ohio State University, Columbus, OH USA; 6 Nicholas School of the Environment, Duke University, Durham, NC USA; 7 Department of Biological Sciences, North Carolina State University, Raleigh, NC 27533 USA; 8 Department of Nutrition, UNC, Chapel Hill, NC USA; 9 Department of OBGYN, Duke University, Durham, NC USA

**Keywords:** Toxic metals, Dietary nutrients, Birthweight, Epidemiology

## Abstract

**Background:**

Cadmium (Cd), lead (Pb) and arsenic (As) are common environmental contaminants that have been associated with lower birthweight. Although some essential metals may mitigate exposure, data are inconsistent. This study sought to evaluate the relationship between toxic metals, nutrient combinations and birthweight among 275 mother-child pairs.

**Methods:**

Non-essential metals, Cd, Pb, As, and essential metals, iron (Fe), zinc (Zn), selenium (Se), copper (Cu), calcium (Ca), magnesium (Mg), and manganese (Mn) were measured in maternal whole blood obtained during the first trimester using inductively coupled plasma mass spectrometry. Folate concentrations were measured by microbial assay. Birthweight was obtained from medical records. We used quantile regression to evaluate the association between toxic metals and nutrients due to their underlying wedge-shaped relationship. Ordinary linear regression was used to evaluate associations between birth weight and toxic metals.

**Results:**

After multivariate adjustment, the negative association between Pb or Cd and a combination of Fe, Se, Ca and folate was robust, persistent and dose-dependent (*p* < 0.05). However, a combination of Zn, Cu, Mn and Mg was positively associated with Pb and Cd levels. While prenatal blood Cd and Pb were also associated with lower birthweight. Fe, Se, Ca and folate did not modify these associations.

**Conclusion:**

Small sample size and cross-sectional design notwithstanding, the robust and persistent negative associations between some, but not all, nutrient combinations with these ubiquitous environmental contaminants suggest that only some recommended nutrient combinations may mitigate toxic metal exposure in chronically exposed populations. Larger longitudinal studies are required to confirm these findings.

**Electronic supplementary material:**

The online version of this article (doi:10.1186/s12889-017-4225-8) contains supplementary material, which is available to authorized users.

## Background

Trace metals including cadmium (Cd), lead (Pb) and arsenic (As) are common environmental contaminants. Main routes of human exposure are ingestion and inhalation of contaminated dust and food [1]. These metals readily enter the food supply [2, 3], bioaccumulate, and target multiple organs. Exposure to these trace metals is implicated in cardiometabolic diseases, including cardiovascular diseases [4, 5], diabetes [4, 6], renal disorders [7, 8] and some types of cancer [9–13]. Some data suggest effects of these metals are sex- or race-specific [14, 15]. In both humans and experimental model systems, fetal exposure to these trace metals is associated with lower birthweight [16–23] and shorter birth length [17, 19, 24–30]. While non-specific, low birthweight is a consistent risk factor for obesity and cardiometabolic disease in adulthood [31–33]. Strategies to reduce or eliminate toxic metal exposure could have widespread implications for fetal health and adult chronic disease.

Dietary manipulation together with environmental management [34], are recommended strategies to mitigate exposure and its downstream health effects in chronically exposed populations. For example, iron (Fe) deficiency in humans has been linked to elevated levels of Cd in both blood and urine, independent of smoking, poverty, age, race, obesity and parity [35]. Deficiencies in essential metals including Cu [36], Fe [37–40], manganese (Mn) [41] and magnesium (Mg) [42–44], and zinc (Zn) [45, 46] have been associated with increased risk of low birth weight and adiposity in some, but not all investigations [47, 48]. In animals, deficiencies in folate (a one-carbon cycle nutrient critical to the generation of S-adenosyl methionine, the universal methyl group donor) reduce As methylation and excretion [49]. This animal data is bolstered by epidemiologic data showing that one-carbon cycle supplementation is associated with re-methylation of interspersed repeat elements [50] and lower As levels in populations chronically exposed to As [51].

Collectively, these data suggest that dietary repletion with essential metals may alleviate trace metal exposure and/or effects. However, associations are inconsistent and, to date, most investigations have only evaluated individual nutrients without examining their combinations or accounting for the non-linear relationship between toxic metals and nutrients [52]. These analyses overcome somes of these limitations by: [1] evaluating the cross sectional relationship between Cd, Pb and As, and eight nutrients (e.g. Fe, selenium [Se], calcium [Ca], Mg, Mn, copper [Cu], Zn and folate), both independently and in combination; and [2] using quantile regression models to account for non-linear effects. We additionally examined whether maternal blood concentrations of either toxic metals or nutrient combinations are associated with offspring weight at birth.

## Methods

### Study participants

Study participants were English or Spanish speaking pregnant women whom, during an 18 month period, between April 2009 and October 2011, were aged ≥18-years of age and attending a prenatal clinic serving Duke University and Durham Regional obstetric facilities [53]. Of 2548 eligible women that were approached, 1700 (66.7%) consented. Women who declined study participation were more likely to be of Asian and Native American (*p* < 0.001) descent, but similar to enrolled women with respect to other characteristics. Cd, As and Pb were measured in whole peripheral blood of the first 310 women enrolled and the present analyses are limited to the 275 mother-infant pairs with complete exposure data for heavy metals, nutrients and birthweight. The distributions of covariates including maternal age at delivery, race/ethnicity, pre-pregnancy body mass index (BMI), education, smoking and gestational age at blood draw and at delivery were comparable in the 275 women and the larger cohort of 1700 (*p* values >0.05).

### Data collection

At enrollment, all participants completed a self- or interviewer-administered questionnaire soliciting information on demographic, reproductive history, lifestyle behaviors, and anthropometric characteristics. Maternal peripheral blood samples were collected at enrollment at median gestational age 12 weeks (Inter-quantile range 8 – 14 weeks) in 10 mL EDTA-treated vaccutainer tubes from which one mL of whole blood was removed for use in these studies and stored at −80 °C. Upon delivery, parturition data were abstracted from medical records.

### Measurement of folate and trace metals

Maternal concentrations of folate, Cd, Pb, As, Fe, Zn, Se, Cu, Ca, Mg and Mn were measured in maternal whole blood. Folate concentrations were measured using a commercial kit, ID-Vit Folic acid (Immundiagnostic-ALPCO; Salem, NH) [54]. Metal concentrations were measured as nanograms per gram (*ng*/*g*) using solution-based inductively coupled plasma mass spectrometry (ICP-MS) methods previously described [55–57]. All standards, including aliquots of the certified NIST 955c, and procedural blanks were prepared by the same process. Maternal metal concentrations were measured using a Perkin Elmer DRC II (Dynamic Reaction Cell) axial field ICP-MS at GeoMed Analtyical [55–58].

### Quality control

To clean sample lines and reduce memory effects, sample lines were sequentially washed with 18.2 MΩ cm resistance (by a Milli-Q water purification system, Millipore, Bedford, Mass., USA) water for 90 s and a 2% nitric acid solution for 120 s between analyses. To monitor and correct for instrumental and procedural backgrounds, procedural blanks were analyzed within each block of 10 samples. Calibration standards included aliquots of 18.2 MΩ cm resistance H_2_O, NIST 955c SRM, and NIST 955c SRM spiked with known quantities of each metal in a linear range from 0.025 to 10 ng/g. Standards were prepared from 1000 mg/L single element standards obtained from SCP Science, USA. Method detection limits (MDLs) were calculated according to the two-step approach using the t99SLLMV method (USEPA, 1993) at 99% CI (*t* = 3.71). To facilitate comparisons with prior studies, trace metal concentrations were converted from *ng*/*g* to *μg*/*dl* based on blood density of 1.035*g*/*ml*. The MDLs yielded values of 0.006, 0.005, and 0.071 μg/dL, for Cd, Pb, and As, respectively. Limits of detection (LOD) were 0.002, 0.002, and 0.022 μg/dL, for Cd, Pb and As, respectively, and limits of quantification (LOQ) (according to Long and Winefordner, 1983) were 0.0007, 0.0006, and 0.0073 μg/dL for Cd, Pb, and As, respectively. The number of samples below the LOD for Cd, Pb, and As were 2, 2, and 1, respectively.

### Assessment of birthweight and covariates

Parturition data were abstracted from medical records by trained personnel after delivery. These data included birthweight (grams), gestational age at birth (weeks) and infant sex (male/female). Infant birthweight was normally distributed and analyzed as a continuous variable. The median values and range for Cd and Pb varied in strata of several covariates. These covariates were explored as potential confounders in the associations between toxic metals and nutrients, as well as birthweight. Covariates included maternal age at delivery (<30, 30–35, and >35 years), race/ethnicity (White, African American, Hispanic), pre-pregnancy BMI (<30/≥30 kg/m^2^), education (< high school, high school graduates/GED, and college graduates), smoking status (non-smoker/smoker), infant sex (male/female), and gestational age at birth (<37/≥37 weeks).

If we observed significant differences in toxic metal levels, we considered these covariates independently as potential confounders. Each individual covariate was retained in the model if its inclusion changed the association under investigation by 10% or more. For analysis of association between birthweight and Cd, Pb or As, a global test was performed to detect significant interaction between these metals and covariates. A priori, we considered maternal smoking and infant sex as potential effect modifiers of the relationship between toxic metals and maternal nutrients, as well as birthweight. We therefore examined associations within strata of these two variables.

### Statistical analyses

Quantile regression models were used to evaluate associations between Cd, Pb and As, individually and eight nutrient concentrations (Fe, Zn, Se, Cu, Ca, Mg, Mn, and total folate) adjusting for maternal delivery age, race/ethnicity, pre-pregnancy BMI, education and smoking. We considered quantile regression because we observed a wedge-shaped relationship between maternal toxic metal and nutrient concentrations (see Additional file 1: Figure S1), which suggested that the magnitude of associations may differ by toxic metal quantile. The quantile regression [59] provided the slope of each nutrient at different quantile levels (*τ*) of the toxic metal (i.e., *τ* = 0.1 to 0.9 by increments of 0.05), adjusting for other nutrients and covariates. For each nutrient, a global test was performed to examine if the nutrient was associated with the toxic metal at any quantile level, and the significance level was Bonferroni-corrected for multiple nutrients and toxic metals, i.e., 0.05/(3 toxic metals ×8 nutrients) = 0.002.

Because nutrients are taken in mixtures and are correlated (see Additional file 2: Table S1), we also considered the aggregate effect of nutrients by computing two multi-nutrient indices: the negative-association nutrient (NAN) measure and the positive-association nutrient (PAN) measure. The NAN index was computed as the sum of the standardized value of Fe, Se, Ca, and folate, which appeared to be negatively associated with toxic metals in the joint analysis. The PAN index was computed as the sum of standardized values of Zn, Cu, Mg and Mn, which appeared to be positively associated with toxic metals in the joint analysis. Standardization before summing assured that the nutrients were pooled together on a comparable scale. For each toxic metal, quantile regressions were conducted treating the toxic metal as the response variable and the two nutrients indices and other covariates as explanatory variables. All results were also Bonferroni-corrected (0.05/(3 toxic metals ×2 nutrient indices) = 0.008) to account for multiple comparisons.

Ordinary linear regression analysis was used to evaluate the association between toxic metals and birthweight, adjusted for maternal age at delivery, race/ethnicity, education, smoking status, pre-pregnancy BMI, gestational age at birth and infant sex, as well as the association between the two nutrient indices (NAN and PAN) and birthweight. Due to the potential non-linear relationship between birthweight and toxic metals (i.e., Additional file 3: Figure S2) and between birthweight and nutrient indices (i.e., Additional file 4: Figure S3), we categorized the toxic metal values and nutrient indices into *low* (i.e., concentration below the 33rd percentile), *moderate* (between the 33rd and 67th percentiles) and *high* (i.e., above the 67th percentile). In addition, because the exposures of Cd and Pb in the NEST cohort are geographically clustered with co-exposure to Cd and Pb [1] (but not among other toxic metals), we modeled the co-exposure effect of Cd and Pb by including the interaction terms between Cd and Pb. We also considered the interactions between toxic metals and nutrient indices to assess if the metal effects on birthweight can be modified by nutrients. Cigarette smoking is a major source of numerous toxic metals including Cd and Pb, and sex-specific effects have been hypothesized previously. Therefore, associations between Cd, Pb or As in relation to birthweight were evaluated in all participants and in strata of prenatal exposure to infant sex and cigarette smoke adjusted by other covariates [30, 60] (a priori *p* = 0.05). Due to the small number of smokers (*n* = 39), we did not estimate associations in this stratum.

## Results

The distributions of individual toxic metals in strata of potential covariates are shown in Table 1. While there was some variation in the range of As by covariate, the median value remained comparable across covariates.

**Table 1:** Characteristics of the 275 study participants

Category		N	Cadmium (μg/dL) quantile: median [IQR*]	Lead (μg/dL) quantile: median [IQR*]	Arsenic (μg/dL) quantile: median [IQR*]
Maternal age at delivery	≤30	178	0.025 [0.012, 0.06]	0.345 [0.153, 0.811]	0.043 [0.038, 0.051]
	(30, 35]	66	0.02 [0.01, 0.039]	0.299 [0.152, 0.726]	0.044 [0.039, 0.051]
	>35	31	0.023 [0.009, 0.033]	0.517 [0.225, 1.407]	0.044 [0.04, 0.048]
Pre pregnancy BMI	≤30	201	0.023 [0.011, 0.053]	0.358 [0.161, 0.83]	0.044 [0.039, 0.052]
	>30	74	0.025 [0.01, 0.042]	0.321 [0.153, 0.844]	0.044 [0.037, 0.049]
Education	College graduate	89	0.014 [0.009, 0.028]	0.275 [0.12, 0.718]	0.043 [0.038, 0.05]
	High school graduate/GED	97	0.029 [0.015, 0.061]	0.268 [0.17, 0.663]	0.043 [0.038, 0.051]
	<high school	89	0.026 [0.012, 0.098]	0.535 [0.235, 1.113]	0.045 [0.041, 0.053]
Smoking status	Non-smoker	235	0.019 [0.01, 0.035]	0.341 [0.15, 0.779]	0.044 [0.038, 0.051]
	Smoker	40	0.051 [0.035, 0.1]	0.404 [0.177, 1.304]	0.045 [0.04, 0.056]
Race/Ethnicity	Black	104	0.031 [0.015, 0.059]	0.339 [0.162, 0.761]	0.043 [0.037, 0.051]
	Hispanic	89	0.02 [0.01, 0.09]	0.517 [0.227, 1.105]	0.044 [0.041, 0.051]
	White	82	0.016 [0.009, 0.029]	0.242 [0.126, 0.716]	0.045 [0.038, 0.051]
Infant sex	Male	132	0.019 [0.01, 0.041]	0.367 [0.145, 0.864]	0.044 [0.039, 0.051]
	Female	143	0.026 [0.012, 0.053]	0.337 [0.178, 0.809]	0.044 [0.038, 0.051]
Gestational age at delivery	<37	25	0.023 [0.009, 0.035]	0.451 [0.151, 0.954]	0.047 [0.043, 0.05]
	> = 37	250	0.024 [0.012, 0.047]	0.341 [0.158, 0.817]	0.043 [0.038, 0.051]

*IQR: interquartile range

### Association between toxic metals and individual nutrients

**Fig. 1:**
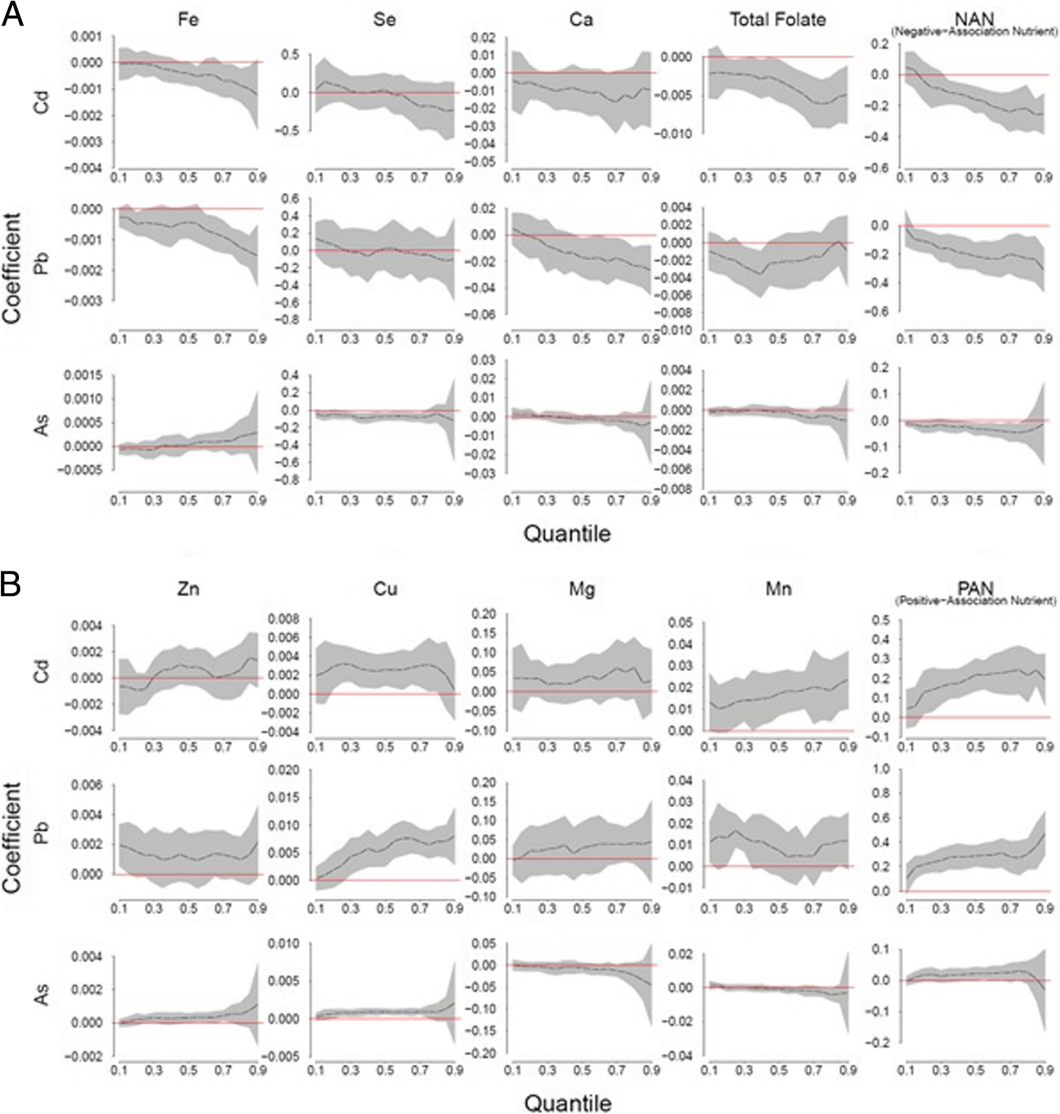
Quantile regression coefficients of 8 nutrients and their indices and toxic metals, Cd, Pb and As. The Y axis indicates the regression coefficients of a nutrient for a given toxic metal; the X axis indicates the quantile of toxic metals. Grey area indicates the 95% simultaneous confidence band for the quantile coefficient estimation; red line indicates regression coefficient = 0

Quantile regression coefficient estimations (*τ*), along with their 95% simultaneous confidence bands [59] adjusted for other nutrients and maternal age, race/ethnicity, pre-pregnancy BMI, education, and smoking, are shown in Fig. 1. We found significant negative associations between Cd and Fe and folate concentrations, and positive associations with Cu and Mn. With the exception of Mn and folate, most regression coefficients were significant in the middle range of Cd. Similarly, negative associations were also found between Pb concentrations and Fe, Ca and folate, while associations with Zn, Cu and Mn were positive. In general, regression coefficients were significant in the upper quantiles of Pb for Fe, Cu and Ca. In contrast, coefficients for folate and Mn were significant in a limited range. For As, we found significant negative associations with Se and folate at only one quantile point, and significant positive associations with Zn and Cu. Coefficients of Se remained relatively constant across quantiles of As, while Zn, Cu and folate had slope estimates further away from zero in upper quantiles of As exposure.

### Association between toxic metals and nutrient mixtures

The last column of Fig. 1 shows quantile regression for the sum of standardized negatively and positively associated indices, NAN and PAN, respectively, in relation to Cd, Pb and As. Cd levels were significantly associated with both nutrition indices (*p*-values <0.0002). In contrast to individual nutrients, where associations were observed for some range of quantiles (i.e. for Fe, τ = 0.55 , 0.7 ~ 0.85), associations with the nutrient index remained significant for most of the quantiles. Similarly, Pb levels were significantly associated with both NAN and PAN indices (*p*-values <0.0002), and the associations remained consistent for all quantiles of Pb. We also observed that As levels remained significantly negatively associated with the NAN index only in a limited range (*τ* = 0.1 ~ 0.15 , 0.4 ~ 0.75) and were no longer significant at the highest quintiles.

### Association between toxic metals, nutrient mixtures and birthweight

Overall, medium level of As exposure were also associated with lower birthweight in all subjects (Table 2A), male infants (Table 2B) and non-smokers (Table 2D). In addition, high level of Cd was found associated with lower birthweight in male infants (Table 2B). In male-only analyses, we also observed significant interaction effects between Cd and PAN as well as between As and PAN. These interaction effects suggested that higher level of PAN might reduce the magnitude of birthweight loss in male infants compared to the baseline levels (i.e.,. low Cd and low PAN and low As and low PAN, respectively).

**Table 2:** Regression coefficients for the associations between toxic metal concentrations and birth weight in males, females and non-smokers

	A. All data (*n* = 272)		B. Male (*n* = 130)		C. Female (*n* = 142)		D. Non-smokers (*n* = 233)	
Caharacteristic	Estimate (Std. Error)	*P*-value	Estimate (Std. Error)	*P*-value	Estimate (Std. Error)	*P*-value	Estimate (Std. Error)	*P*-value
(Intercept)	**−4721.71** ^*^ **(693.49)**	<0.001	**−3782.55 (1161.8)**	0.002	**−4546.38 (1075.15)**	<0.001	**−4251.48 (869.47)**	<0.001
Moderate Cd	−125.97 (160.05)	0.432	−264.59 (264.87)	0.321	−70.98 (247.9)	0.775	−111.12 (172.28)	0.520
High Cd	−382.74 (210.06)	0.070	**−812.25 (346.1)**	0.021	−201.44 (356.57)	0.573	−290.2 (264.06)	0.273
Moderate Pb	−68.42 (160.65)	0.671	−225.21 (359.43)	0.533	35.51 (199.73)	0.859	−22.5 (173.71)	0.897
High Pb	−120.42 (200.95)	0.550	11.01 (395.46)	0.978	−58.88 (262.59)	0.823	−191.24 (223.77)	0.394
Moderate As	**−366.5 (175.2)**	0.038	**−870.69 (372.09)**	0.022	−180.59 (218.97)	0.412	**−464.29 (202.74)**	0.023
High As	72.82 (139.83)	0.603	−310.42 (242.22)	0.204	199.52 (195.24)	0.309	163.61 (161.11)	0.311
Moderate Cd:oderate Pb	68.5 (164.92)	0.678	3.27 (290.12)	0.991	23.69 (254.14)	0.926	95.5 (176.05)	0.588
High Cd:Moderate Pb	129 (200.79)	0.521	477.08 (428.43)	0.269	−63.06 (312.3)	0.84	−68.99 (277.85)	0.804
Moderate Cd:High Pb	403.84 (226.22)	0.076	65.54 (368.9)	0.859	**785.05 (363.23)**	0.033	322.81 (251.94)	0.202
High Cd:High Pb	113.2 (220.94)	0.609	47.79 (413.45)	0.908	345.83 (357.83)	0.336	−68.44 (301.77)	0.821
Moderate NAN	69.17 (171.44)	0.687	−272.91 (274.51)	0.323	309.38 (332.21)	0.354	−24.77 (187.92)	0.895
High NAN	133.53 (179.46)	0.458	62.14 (319.67)	0.846	48.16 (248.6)	0.847	104.1 (192.18)	0.589
Moderate PAN	−106.41 (173.19)	0.540	−527.03 (279.37)	0.063	154.08 (286.47)	0.592	−16.12 (188.07)	0.932
High PAN	−306.32 (196.91)	0.121	**−736.08 (353.06)**	0.04	−171.63 (276.41)	0.536	−271.49 (213.82)	0.206
Moderate Cd:Moderate NAN	−15.56 (187.42)	0.934	−83.86 (330.37)	0.8	−52.55 (276.51)	0.85	−19.69 (205.13)	0.924
High Cd: Moderate NAN	103.44 (190.11)	0.587	433.38 (344.66)	0.212	−114.8 (278.45)	0.681	220.18 (219.16)	0.316
Moderate Cd:High NAN	99.37 (213.17)	0.642	−197.68 (361.34)	0.586	317.79 (318.09)	0.320	42.21 (230.92)	0.855
High Cd:High NAN	170.97 (226.96)	0.452	316.61 (462.66)	0.496	77.43 (309.97)	0.803	190.84 (278.84)	0.495
Moderate Cd:Moderate PAN	55.91 (194.67)	0.774	307.21 (334.27)	0.361	12.31 (270.51)	0.964	63.2 (212.11)	0.766
High Cd:Moderate PAN	361.59 (188.45)	0.056	368.06 (366.16)	0.318	458.2 (281.48)	0.107	456.64 (233.05)	0.052
Moderate Cd:High PAN	202.64 (230.04)	0.379	**894.08 (404.05)**	0.030	−155.77 (322.56)	0.630	263.34 (250.81)	0.295
High Cd:High PAN	**477.25 (229.66)**	0.039	711.43 (445.54)	0.114	481.47 (334.96)	0.154	449.77 (286.85)	0.119
Moderate Pb:Moderate NAN	−187.78 (187.41)	0.317	170.59 (340.91)	0.618	−351.75 (296.46)	0.238	−224.7 (210.09)	0.286
High Pb:Moderate NAN	−153.04 (222.77)	0.493	−131.75 (396.94)	0.741	−542.56 (339.57)	0.113	−145.29 (255.15)	0.570
Moderate Pb:High NAN	−126.16 (204.89)	0.539	118.2 (388.7)	0.762	−92.68 (292.05)	0.752	−125.5 (231.1)	0.588
High Pb:High NAN	6.28 (235.71)	0.979	−194.48 (469.11)	0.68	−142.03 (353.1)	0.688	3.89 (282.4)	0.989
Moderate Pb:Moderate PAN	30.45 (179.31)	0.865	43.46 (340.04)	0.899	−124.46 (281.42)	0.659	−43.8 (206)	0.832
High Pb:Moderate PAN	−109.95 (226.29)	0.628	21.11 (404.4)	0.958	−210.73 (364.93)	0.565	−62.16 (267.16)	0.816
Moderate Pb:High PAN	210.19 (199.96)	0.294	200.26 (365.37)	0.585	266.68 (278.08)	0.340	210.73 (223.78)	0.348
High Pb:High PAN	−236.39 (263.92)	0.371	11.09 (464.4)	0.981	−416.52 (406.03)	0.308	−68.75 (321.64)	0.831
Moderate As:Moderate NAN	131.74 (201.75)	0.514	284.7 (390.7)	0.468	34.85 (275.87)	0.900	313.29 (229.55)	0.174
High As:Moderate NAN	−272.79 (213.48)	0.203	−274.26 (368.65)	0.459	−199.63 (316.75)	0.530	−243.52 (263.43)	0.356
Median As:High NAN	42.14 (236.38)	0.859	215.27 (442.65)	0.628	5.1 (326.28)	0.988	202.7 (273.88)	0.46
High As:High NAN	**−430.35 (210.5)**	0.042	−548.28 (352.69)	0.124	−163.32 (326.11)	0.618	−464.49 (236.44)	0.051
Median As:Median PAN	177.57 (180.43)	0.326	**683.69 (326.62)**	0.039	24.95 (251.2)	0.921	162.32 (201.93)	0.423
High As:Median PAN	81.74 (202.68)	0.687	**710.58 (315.78)**	0.027	−394.93 (305.35)	0.199	7.11 (244.53)	0.977
Median As:High PAN	195.33 (218.9)	0.373	294.86 (394.46)	0.457	206.5 (318.7)	0.519	119.09 (244.02)	0.626
High As:High PAN	158.09 (231.82)	0.496	435.1 (411.65)	0.294	−23.33 (326.51)	0.943	63.95 (256.37)	0.803

*: Coefficients that are statistically significant are shown in bold. Negative-association nutrient (NAN), obtained by the sum of the standardized values of Fe, Se, Ca and total folate. Positive-association nutrient (PAN), obtained by the sum of the standardized values of Zn, Cu, Mg and Mn. Models adjusted for maternal age, ethnicity, cigarette smoking, educational attainment, gestational age at delivery and blood draw and sex

There were also significant metal and nutrient interacitons observed in all-subject analysis, including between Cd and PAN (where higher PAN levels reduced the magnitude of birthweight loss) as well as between As and NAN (for which, though high As and high NAN individually are positively associated with birthweight, the existence of both leads to birthweight loss).

We did not find significant co-exposure effects between Pb and Cd except in the female-only analysis (Table 2C), where we observed that female infants with moderate Cd and high Pb exposure tend to have higher birthweight compared to the baseline group of low Cd and low Pb.

## Discussion

We evaluated associations between Cd, Pb and As, and eight nutrients in pregnant women, and determined if exposure to these metals were associated with lower birthweight. We found that higher levels of a combination comprising Fe, Ca, Se and folate was robustly related to lower Pb and Cd, regardless of concentration of these toxic metals, whereas the negative relationships with single nutrients were within very narrow ranges of exposure. Surprisingly, higher levels of Cu, Zn, Mg and Mn were associated with higher levels of Pb and Cd. With the exception of recent data supporting positive associations between Mn and Cd [61], these findings are despite accumulated animal data and human single nutrient data to the contrary. Finally, exposure to Cd and As were negatively associated with birthweight as would be expected; but these associations were modified by select nutrient combinations. These findings are consistent with previous studies demonstrating that dietary repletion with Fe, Ca, Se and folate can mitigate exposure to Cd and Pb. However, our findings suggesting that Cu, Zn, Mg and Mn are positively associated with Cd and Pb levels contrast with previous studies that form the basis for existing intervention guidelines as they suggest these minerals may exacerbate exposure.

Our data showing negative, but weak and inconsistent associations between Pb, As or Cd and single nutrients are consistent with these divalent cations Fe^2+^ or Ca^2+^ competitively displacing Cd^2+^ in transmetallation reactions. In animals, Fe reduces intestinal absorption of Cd [35]. These animal data are corroborated by human studies showing that Fe-deficiency is consistently associated with higher blood and urine Cd levels, independent of smoking, poverty, age, race, obesity and parity [35]. Our data however contrast with in vitro evidence that show >50% lower Cd levels with Zn and Mn treatment, and negative relationships between Zn, Cu and Mg with Cd [62]. These data provide early evidence that some essential metals, including Mn [63] may not mitigate exposure and effects of these metals. Specific mineral combinations that mitigate exposure may depend on the underlying nutritional status and may therefore vary by population exposed to Pb or Cd.

One-carbon cycle nutrients are critical in generating S-adenosyl methionine, key in the clearance of As [64]. In mice [65] and humans [66–69], higher circulating one-carbon nutrients are associated with a lower body burden of inorganic As. Depletion of one-carbon cycle nutrients reduces As excretion and methylation [49, 70], whereas their repletion lowers blood As levels [51]. In 6-year old As-exposed children, one-carbon cycle supplementation was associated with re-methylation of interspersed repeat elements [50] and lower As levels [51]. Although fewer studies have been conducted on Pb or Cd, negative associations with folate or vitamin B12 have been reported [71]. Our data provides evidence for negative associations between folate in combination with Fe, Ca and Se, and Cd or Pb levels.

Consistent with previous studies, we also found that prenatal exposure to Cd or As is associated with lower birthweight [16–20]. This adverse birth outcome increases the risk of rapid adiposity gain in young children; a consistent risk factor for cardiometabolic impairment in adulthood [72–78]. In adult cross-sectional studies, elevated Cd, Pb and As have been associated with cardiometabolic risk markers [79–85]. While metabolic syndrome is not clinically discernible in young children [86], individual cardiometabolic risk markers that include central adiposity, elevated systolic blood pressure and elevated levels of fasting insulin and/or glucose, triglycerides, cholesterol, accelerated adiposity, [87–91, 76–78, 92–95] sometimes without overt obesity [96], predict metabolic syndrome, atherosclerosis, diabetes and hypertension in adulthood [97–104]. While not confirmed by others [47], increased adiposity and higher insulin have been associated with deficiency of Cu [36], Fe [37–40], Mn [41], Mg [42–44] and Zn [45, 46]. Thus, the low birthweight that is associated with early exposure to these toxic metals supports the developmental origins of these cardiometabolic diseases, and may portend, and/or contribute to the increase in incidence of these diseases.

While these data suggest that some nutrients may mitigate toxic metal exposure and effects, mechanisms are poorly understood. In animal and in vitro models, ferritin reduces intestinal absorption of Cd [35] and Pb. Mn also shares transporters with Cd and Pb in vivo and in vitro. Cd induces oxidative stress via decreasing cellular antioxidant capacity, increases lipid peroxidation, and depletes glutathione and protein-bound sulfhydryl groups [105–107]. Because the target organs for these metals such as the kidney and liver also play a critical role in the maintenance of blood glucose levels [108–111], associations between these toxic metals and low birthweight that is often followed by accelerated adiposity gains and insulin resistance, are to be expected.

Our findings should be interpreted in the context of the study limitations. The relatively small sample size limited our ability to assess subgroup effects, and particularly higher order interactions (e.g. by combined maternal smoking, nutrient combinations and race/ethnicity), which may inform public health interventions. In addition, while we anticipate that some nutrient combinations may mitigate toxic metal exposure, cause-and-effect cannot be inferred in this cross-sectional study. Furthemore, we assessed maternal nutrient concentrations at a single time-point, yet these nutritional markers may vary throughout gestation, as the physiologic changes that occur during pregnancy may impact levels of toxic metals in maternal blood. For example, the increase in erythrocytes and plasma with advancing gestation may lower levels, presumably due to hemodilution, whereas essential elements may increase, in part due to increased supplementation. In support, a Canadian study reported that while Cd levels did not change, Pb levels decreased over the course of pregnancy, while Mn levels increased during the same period [63]. Thus, given our specimens were collected during a short gestational window (median 12 weeks, IQR 8–14 weeks), the full effect of toxic metal and nutrients on birthweight may only be partially realized. However, sensitivity analyses showed that restricting analysis to women with gestational age > 14 weeks at blood draw did not alter our findings. Despite these limitations, our study has several strengths including the multiethnic composition of the cohort and our ability to examine multiple nutrients to mitigate exposure and effects very early in gestation, when many metabolic set points are established.

## Conclusions

In summary, we provide early data suggesting that a combination of Fe, Ca, Se and folate is negatively associated with Cd and As exposure, while Cu, Zn, Mg and Mn may exacerbate exposure to these toxic compounds. We also confirmed negative associations between birthweight and toxic compounds, Cd and As. Larger studies are required to identify other nutrients, which in combination, may mitigate exposure to these ubiquitous toxic metals in exposed populations.

## Additional files

Additional file 1: Figure S1. Dashed black line is the ordinary least square regression fitted line which is flat indicating weak association between logCd and Fe at mean level. Solid red, yellow and blue line are the quantile regression fitted line on quantile of logCd at 90th quantile 50th quantile and 10th quantile respectively. (PNG 61 kb)

Additional file 2: Table S1. Spearman correlation among all nutrients. (DOC 32 kb)

Additional file 3: Figure S2. Boxplot of infant birthweights on different levels of toxic metals. Toxic metals are classified into 3 levels: Low (33.3rd quantile and below), Moderate (33.3rd to 66.7th quantile) and High (66.7th quantile above). The results suggest a potential non-linear relationship between birthweight and Cd as well as between birthweight and As. (PNG 28 kb)

Additional file 4: Figure S3. Boxplot of infant birth weight on different levels of nutrients indices, i.e., NAN and PAN. Nutrients indices are classified into 3 levels: Low (33.3rd quantile and below), Moderate (33.3rd to 66.7th quantile) and High (66.7th quantile above). The plots suggest a potential non-linear relationship between birthweight and NAN as well as between birthweight and PAN. (PNG 24 kb)

## Abbreviations

As: Arsenic
BMI: Body mass index
Cd: Cadmium
Ca: Calcium
Cu: Copper
ICP-MS: Inductively coupled plasma mass spectrometry
Fe: iron
Pb: Lead
LOD: Limits of detection
LOQ: Limits of quantification
Mg: magnesium
Mn: Manganese
NAN: Negative-association nutrient
PAN: Positive-association nutrient
Se: selenium
Zn: zinc
