# Supplementary figures and images for: Maternal blood cadmium, lead and arsenic levels, nutrient combinations, and offspring birthweight
Source: BMC Public Health. 2017 Apr 24;17:354. doi: 10.1186/s12889-017-4225-8 (PMC5402649; doi:10.1186/s12889-017-4225-8)

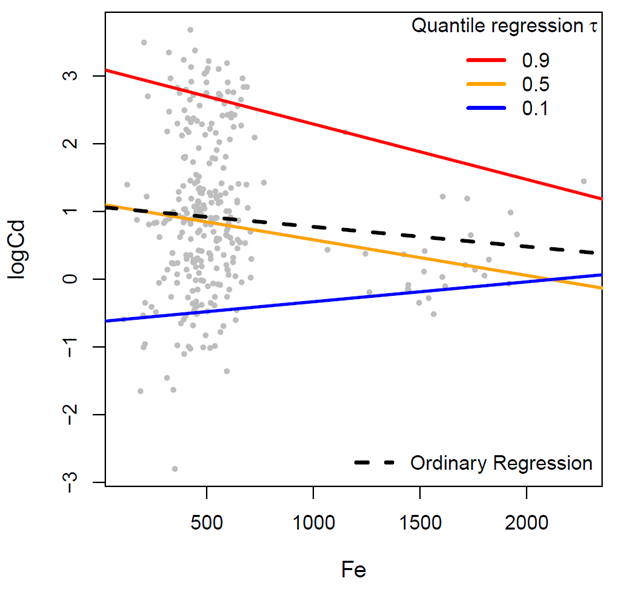

Supplement: Supplementary file 1 — Dashed black line is the ordinary least square regression fitted line which is flat indicating weak association between logCd and Fe at mean level. Solid red, yellow and blue line are the quantile regression fitted line on quantile of logCd at 90th quantile 50th quantile and 10th quantile respectively. (PNG 61 kb) [file 12889_2017_4225_MOESM1_ESM.png]

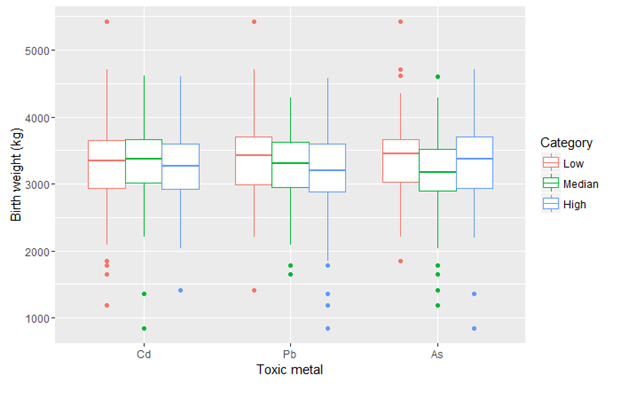

Supplement: Supplementary file 3 — Boxplot of infant birthweights on different levels of toxic metals. Toxic metals are classified into 3 levels: Low (33.3rd quantile and below), Moderate (33.3rd to 66.7th quantile) and High (66.7th quantile above). The results suggest a potential non-linear relationship between birthweight and Cd as well as between birthweight and As. (PNG 28 kb) [file 12889_2017_4225_MOESM3_ESM.png]

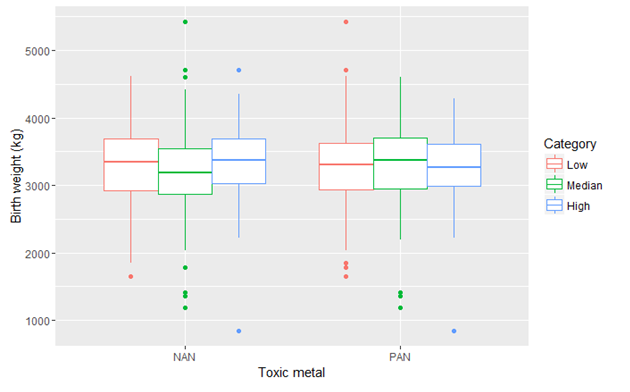

Supplement: Supplementary file 4 — Boxplot of infant birth weight on different levels of nutrients indices, i.e., NAN and PAN. Nutrients indices are classified into 3 levels: Low (33.3rd quantile and below), Moderate (33.3rd to 66.7th quantile) and High (66.7th quantile above). The plots suggest a potential non-linear relationship between birthweight and NAN as well as between birthweight and PAN. (PNG 24 kb) [file 12889_2017_4225_MOESM4_ESM.png]
