# Supplementary material for: Maternal blood cadmium, lead and arsenic levels, nutrient combinations, and offspring birthweight
Source: BMC Public Health. 2017 Apr 24;17:354. doi: 10.1186/s12889-017-4225-8 (PMC5402649; doi:10.1186/s12889-017-4225-8)
Supplement: Supplementary file 2 — Spearman correlation among all nutrients. (DOC 32 kb) [file 12889_2017_4225_MOESM2_ESM.doc]

Supplement Table 1:

|  | Fe | Zn | Se | Cu | Ca | Mg | Mn | Folate |
| --- | --- | --- | --- | --- | --- | --- | --- | --- |
| Fe | 1.000 | 0.301 | 0.431 | -0.035 | 0.103 | 0.405 | 0.537 | 0.109 |
| Zn | 0.301 | 1.000 | 0.031 | 0.042 | -0.126 | 0.324 | 0.071 | 0.196 |
| Se | 0.431 | 0.031 | 1.000 | 0.305 | 0.134 | 0.064 | 0.494 | -0.014 |
| Cu | -0.035 | 0.042 | 0.305 | 1.000 | 0.329 | -0.019 | 0.228 | 0.047 |
| Ca | 0.103 | -0.126 | 0.134 | 0.329 | 1.000 | 0.215 | 0.116 | -0.187 |
| Mg | 0.405 | 0.324 | 0.064 | -0.019 | 0.215 | 1.000 | 0.286 | 0.250 |
| Mn | 0.537 | 0.071 | 0.494 | 0.228 | 0.116 | 0.286 | 1.000 | 0.056 |
| Folate | 0.109 | 0.196 | -0.014 | 0.047 | -0.187 | 0.250 | 0.056 | 1.000 |

Spearman correlation among all nutrients.
